# Dimorphite-DL: an open-source program for enumerating the ionization states of drug-like small molecules

**DOI:** 10.1186/s13321-019-0336-9

**Published:** 2019-02-14

**Authors:** Patrick J. Ropp, Jesse C. Kaminsky, Sara Yablonski, Jacob D. Durrant

**Affiliations:** Department of Biological Sciences, University of Pittsburgh, 4249 Fifth Avenue, Pittsburgh, PA 15260 USA

**Keywords:** Ionization, pH, Protonation, Modeling, Virtual screening, Drug discovery

## Abstract

Small-molecule protonation can promote or discourage protein binding by altering hydrogen-bond, electrostatic, and van-der-Waals interactions. To improve virtual-screen pose and affinity predictions, researchers must account for all major small-molecule ionization states. But existing programs for calculating these states have notable limitations such as high cost, restrictive licenses, slow execution times, and poor modularity. Here, we present dimorphite-DL 1.0, a fast, accurate, accessible, and modular open-source program for enumerating small-molecule ionization states. Dimorphite-DL uses a straightforward empirical algorithm that leverages substructure searching and draws on a database of experimentally characterized ionizable molecules. We have tested dimorphite-DL using several versions of Python and RDKit on all major operating systems. We release it under the terms of the Apache License, Version 2.0. A copy is available free of charge from http://durrantlab.com/dimorphite-dl/.

**Electronic supplementary material:**

The online version of this article (10.1186/s13321-019-0336-9) contains supplementary material, which is available to authorized users.

## Introduction

Structure-based virtual screening (VS) predicts the geometry of a small molecule bound to its receptor (i.e., the docked pose) and maps that geometry to a score that correlates with affinity. Ligand protonation can impact electrostatic, hydrogen-bond, and van-der-Waals interactions between the ligand and receptor [1], potentially affecting both VS steps. Many ligands adopt multiple protonation states, or protomers. Protomers encompass ionization forms, which involve the gain or loss of a proton, and tautomeric forms, which involve the intramolecular transfer of a proton from one ligand atom to another [1]. Transitions between protomers (e.g., via proton uptake or release [2]) often accompany binding [3]. As most small-molecule drugs are ionizable [4, 5], accurate VS must consider the protomer that best complements the binding pocket [6, 7].

Predicting acid ionization constants (pK_a_) is a critical first step. Empirical approaches such as linear free-energy calculations [8], quantitative structure–property relationships, and database similarity searches perform this prediction quickly and so are well suited for processing large compound libraries [5]. In contrast, quantum mechanical methods are slower and not necessarily more accurate [5].

After using predicted pK_a_ values to identify all possible ionization forms, the next step is to discard those forms that are rare. Ligands interconvert between all ionization states in solution, but the pH determines which state is favored. For example, at physiological pH (7.4), 99.96% of 3-chloropropanoic acid (pK_a_ = 4.0 [9]) exists in the deprotonated form, 3-chloropropanoate. It is reasonable to ignore the rare protonated form when performing a VS with limited computational resources. In contrast, 44.27% of 2,2,2 trifluoroethane 1 thiol (pK_a_ = 7.3 [10]) exists in the deprotonated form at physiological pH. Proper small-molecule preparation should consider both the deprotonated and protonated forms of this compound.

Enumerating major small-molecule ionization states can improve virtual-screen predictivity, but available programs for performing this task are generally too expensive, have restrictive licenses, are too slow for use in high-throughput contexts, predict a single state rather than all major states, and/or cannot be easily incorporated into broader drug-discovery pipelines. There is a need for a fast, accurate, accessible, and modular open-source alternative. We have developed a computer program called dimorphite-DL to address this need. We have tested dimorphite-DL using several versions of Python (2.7.13, 3.6.3, 3.6.5, and 3.6.6) and RDKit (2016.09.2, 2018.03.1, and 2018.03.4) on macOS High Sierra 10.13.4, Ubuntu 18.04.1 LTS, and Windows 10 Home 1709. We release it under the terms of the Apache License, Version 2.0. A copy is available free of charge from http://durrantlab.com/dimorphite-dl/.

## Implementation

### A set of compounds with experimental pK_a_ values

We assembled a set of 1938 small molecules with single, diverse ionizable sites and mostly experimentally determined pK_a_ values. To the extent possible, we limited our search to pK_a_ values measured in neutral aqueous solutions near room temperature (e.g., between 23 and 27 °C). Sources of experimental data included PubChem^®^, a chemical database provided by the NIH’s National Library of Medicine; iBonD 2.0, the Internet Bond-Energy Databank provided by Tsinghua and Nankai Universities [11]; Reaxys, a chemical database provided by Elsevier Life Sciences IP Limited; and a published work by Lee et al. [12] that describes monoprotic small molecules. We also separately considered a set of 78 phosphates and phosphonates, which can lose up to two protons.

We performed limited data filtering to improve applicability and accuracy. For example, we removed some molecules with multiple disconnected fragments (e.g., salts) and chiral centers. If a given molecule included multiple experimental pK_a_ values that spanned a range greater than 1.0, we assumed experimental uncertainty and discarded the molecule. Otherwise, we averaged the available pK_a_ values. Per previous studies [12], we generally only considered molecules with measured pK_a_ values between − 1.74 (H_3_O^+^) and 15.7 (H_2_O). In total, 98.8% of the pK_a_ values in our database met this criterion. To ensure proper coverage, we included nine sulfonates and sulfates with pK_a_ values less than − 1.74. We also included fourteen molecules with pK_a_ values greater than 15.7: four non-phenol alcohols, three amides, and seven molecules with protonated but uncharged aromatic nitrogen atoms. Finally, to ensure that nitro-group oxygen atoms are always deprotonated, we assigned a very negative, arbitrary pK_a_ value (− 1000.0) to this group.

We grouped these compounds by ionizable moiety and constructed pK_a_ histograms for each group. Although chemical features beyond the moiety itself (e.g., neighboring electronegative groups) do impact pK_a_, our analysis provided a typical pK_a_ range for each ionizable site. In some cases, visual inspection of the histograms led us to reconsider some moiety definitions. For example, the distribution of amide pK_a_ values was initially bimodal. By separating amides from nitrogens bonded to electronegative atoms, we divided this group into two chemically distinct populations.

Ultimately, we settled on 38 ionizable substructures. In some cases, a given moiety could belong to two such categories. For example, every amide contains an amine group. To uniquely assign each moiety to a given categorization, we prioritized the substructures. Atoms belonging to high-priority substructures that cover more atoms (e.g., amides) were not considered when subsequently searching for lower-priority substructures (e.g., amines).

For each set of compounds matching one of these substructures, we calculated the mean (µ) and standard deviation (σ) of the associated pK_a_ values (Table 1). In the case of the phosphates and phosphonates, each moiety was associated with two separate pK_a_ means and standard deviations (µ_1_ and σ_1_; and µ_2_ and σ_2_), one for each ionizable proton. A range of reasonable pK_a_ values for each moiety, range_PKA_, is given by [µ − *n*σ, µ + *n*σ], where *n* is a user-defined parameter we call the “pK_a_ precision factor.”

**Table 1:**
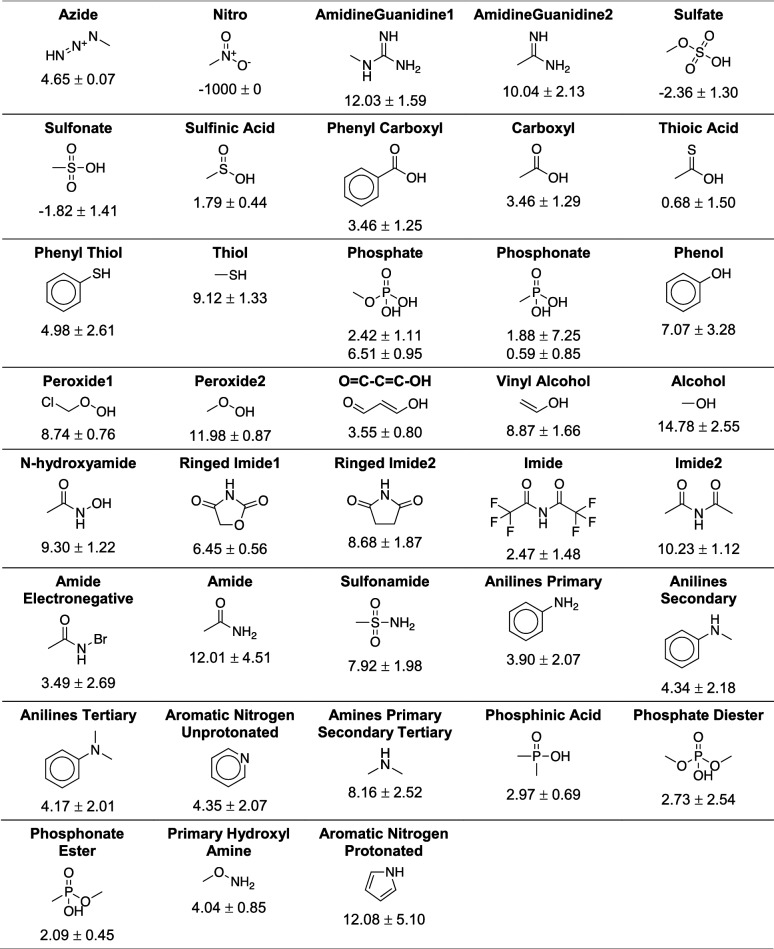
The 38 ionizable dimorphite-DL substructures in order of decreasing priority from left to right, with representative compounds

Exact substructure definitions are given in Additional file 1: Table S1. The pK_a_ range is the average of all associated pK_a_ values in the database, plus or minus the standard deviation

### Predicting ionization states

Dimorphite-DL 1.0 uses the µ and σ values associated with each ionizable moiety to predict small-molecule ionization states for a given pH range. It accepts the following user inputs:

1. A small-molecule library in SMILES format [13], with each compound SMILES on its own line. Alternatively, the user can provide a single SMILES as a command-line parameter.
2. The pK_a_ precision factor to use when estimating moiety pK_a_ ranges (*n*, 1.0 by default).
3. The minimum pH to consider (*pH*_*min*_, 6.4 by default).
4. The maximum pH to consider (*pH*_*max*_, 8.4 by default).

For each molecule, dimorphite-DL uses RDKit [14], an open-source cheminformatics library, to search for the 38 ionizable substructures described above (Table 1 and Additional file 1: Table S1). The same prioritization scheme ensures that any given atom is assigned to at most only one category. The program outputs protonated/deprotonated SMILES, as appropriate for the user-specified pH range.

**Fig. 1:**
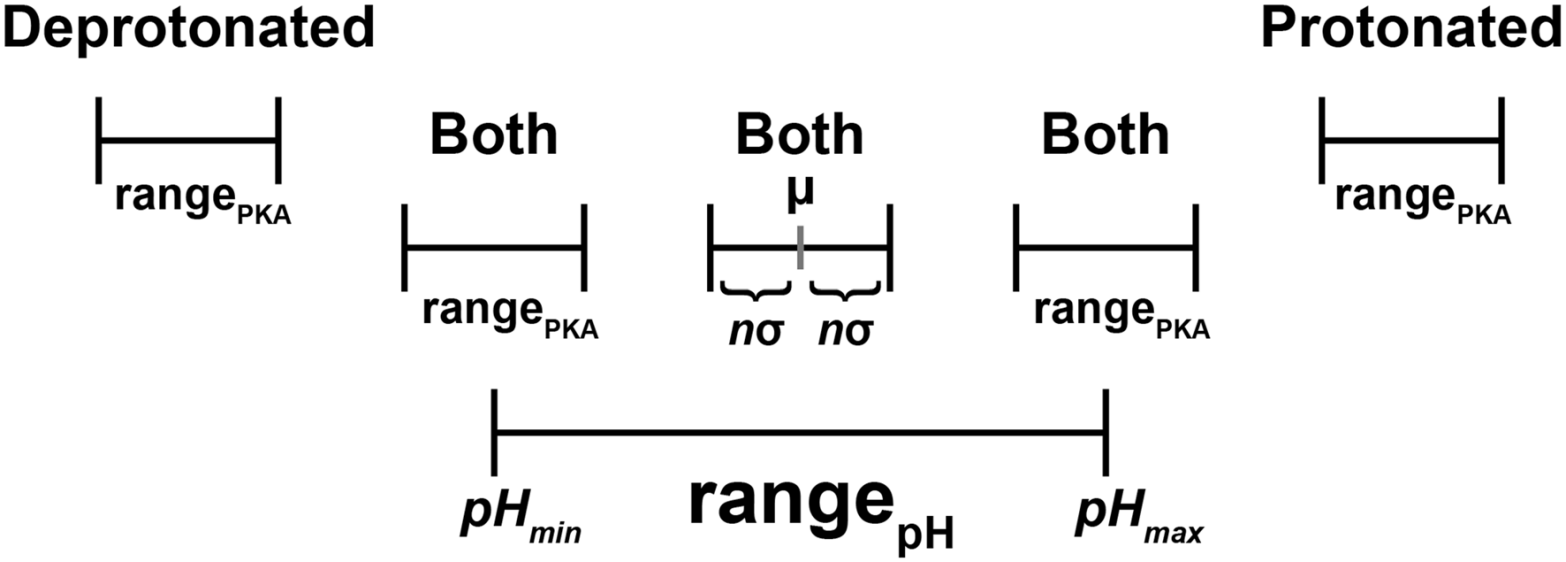
A schematic representation of the dimorphite-DL approach. Each ionizable moiety is associated with a pK_a_ range (range_PKA_) defined by three parameters: µ, σ, and *n*. The user specifies a pH range (range_pH_) and pK_a_ precision factor (*n*; default: 1.0). The mean (µ) and standard deviation (σ) associated with each moiety are derived from the database of small molecules with experimentally characterized pK_a_ values. If range_PKA_ is entirely less than range_pH_, dimorphite-DL outputs a deprotonated molecule. If range_PKA_ is entirely greater than range_pH_, dimorphite-DL outputs a protonated molecule. If range_PKA_ and range_pH_ overlap, dimorphite-DL outputs both deprotonated and protonated molecules

Dimorphite-DL does not calculate pK_a_ values explicitly. Rather, for each categorized moiety, it takes one of three actions (Fig. 1) based on a moiety-specific pK_a_ range, range_PKA_ = [µ − *n*σ, µ + *n*σ], and a user-defined pH range, range_pH_ = [*pH*_*min*_, *pH*_*max*_]:

1. If µ + *n*σ < *pH*_*min*_ (i.e., the entirety of range_PKA_ is less than the entirety of range_pH_), the moiety is deprotonated.
2. If *pH*_*max*_ < µ − *n*σ (i.e., the entirety of range_PKA_ is greater than the entirety of range_pH_), the moiety is protonated.
3. If range_PKA_ and range_pH_ overlap, two distinct small-molecule models are generated, with the moiety protonated and deprotonated, respectively.

### Substructure identification using SMARTS

Dimorphite-DL uses SMARTS (SMILES arbitrary target specification [15]) to determine which atoms in a given molecule belong to one of the 38 ionizable substructures. SMARTS is a language for molecular subgraph isomorphism and pattern matching that is an extension of the popular SMILES format [13]. SMILES describes a molecule, but SMARTS describes a molecular pattern (e.g., a substructure). All SMILES strings are valid SMARTS strings, but SMARTS additionally allows for variable atom and bond specifications. A given SMARTS string can thus represent multiple related chemical structures.

SMARTS pattern recognition requires standardized input molecules. Dimorphite-DL attempts to standardize all input SMILES strings automatically. For example, N[N+]#N, N=[N+]=N, NN#N, and N=N=N are all recognized as azides. Both [N+]([O−])=O and N(=O)=O are recognized as nitro groups. And aromatic rings are recognized regardless of whether the input SMILES string describes aromatic bonds (e.g., both Oc1ccccc1 and OC1=CC=CC=C1 are recognized as phenols).

The Additional file 1 includes the SMARTS representations of the 38 substructures, as well as the calculated µ and σ values (Additional file 1: Table S1). The Additional file 1 also describes how dimorphite-DL independently handles phosphate and phosphonate groups.

## Results and discussion

Dimorphite-DL 1.0 is a fast, accessible, open-source Python program for predicting small-molecule ionization states. As a simple illustration of the importance of accounting for alternate ionization states, consider the local anesthetic lidocaine. The pK_a_ of lidocaine is 8.01 [16], so both the charged protonated- and deprotonated-amine forms are prevalent at physiological pH. Computational evidence suggests that both forms bind the sodium channel NavPas, preventing cell depolarization [17]. But the two protomers have different poses [17], and charged lidocaine binds with higher affinity [17]. It is thus critical to account for both ionization states. Dimorphite-DL applied to lidocaine successfully predicted both forms.

### The dimorphite-DL approach

Dimorphite-DL uses a substructure-based empirical algorithm to quickly prepare large compound libraries for virtual screening (VS). Importantly, it is not limited to identifying a single ionizable state per molecule. Rather, it can generate multiple states as appropriate for a given pH range, thus increasing the chance of identifying the most binding-compatible state.

Because distant chemical groups can impact proton dissociation, the ideal program for enumerating ionizable states would calculate pK_a_ values in the context of the whole molecule. But creating such a program is challenging. Methods that consider whole-molecule contexts, such as quantum mechanical approaches, are too slow for high-throughput use. Surprisingly, their pK_a_ calculations are not necessarily more accurate than those of simpler algorithms [5]. Empirical methods such as dimorphite-DL are faster, but they draw on chemical databases that cannot account for all possible chemical contexts. Dimorphite-DL predicts ionization states by considering only at most a few atoms adjacent to each ionizable moiety. In compounds with multiple ionizable sites, each site is considered independently. Our algorithm thus does not account for interactions between sites or other electronic effects.

To compensate for this limitation, we associate 38 ionizable moieties with pK_a_ *ranges* rather than *point values*. We derive ranges from the experimental pK_a_ values of 1938 small molecules (see Materials and Methods). For each moiety, range_PKA_ = [µ − *n*σ, µ + *n*σ], where µ and σ are the mean and standard deviation of the associated experimental pK_a_ values, respectively; and *n* is a user-defined “pK_a_ precision factor”. Protonation is assigned based on the overlap between range_PKA_ and the user-specified pH range (Fig. 1).

### Dimorphite-DL accuracy: correct, excessive, and incorrect predictions

Defining terms will allow us to better describe the accuracy of Dimorphite-DL predictions. Consider a user-defined pH range, range_pH_ = [*pH*_*min*_, *pH*_*max*_], and a compound with an experimentally determined pK_a_ value. Further assume that the compound can lose at most one proton. Applying dimorphite-DL to this compound can have one of three outcomes:

1. Dimorphite-DL predicts the correct state
  - pK_a_ < *pH*_*min*_, and dimorphite-DL deprotonates the compound
  - pK_a_ > *pH*_*max*_, and dimorphite-DL protonates the compound
  - *pH*_*min*_ ≤ pK_a_ ≤ *pH*_*max*_, and dimorphite-DL generates both deprotonated and protonated forms
2. Dimorphite-DL predicts an excess state (i.e., two states when only one is appropriate)
  - pK_a_ < *pH*_*min*_ or pK_a_ > *pH*_*max*_, but dimorphite-DL generates both deprotonated and protonated forms
3. Dimorphite-DL predicts the incorrect (or incomplete) state
  - pK_a_ < *pH*_*min*_, but dimorphite-DL protonates the compound
  - pK_a_ > *pH*_*max*_, but dimorphite-DL deprotonates the compound
  - *pH*_*min*_ ≤ pK_a_ ≤ *pH*_*max*_, and dimorphite-DL either deprotonates or protonates the compound (not both)

We distinguish between “excess-state” and “incorrect-state” outcomes because they differ in their consequences. If dimorphite-DL predicts an excess state, it needlessly expands the compound library and increases the computational expense of subsequent VS. But if it predicts an incorrect or incomplete state, VS accuracy may suffer because a relevant state is never generated.

### The influence of the pK_a_ precision factor on accuracy

Recall that each moiety has an associated range_PKA_ ([µ − *n*σ, µ + *n*σ]) determined in part by the user-specified pK_a_ precision factor, *n*. To assess the influence of this factor on accuracy, we evaluated the compounds in our database using different values of *n* (Table 2), always over the default range_pH_ = [6.4, 8.4]. As *n* increases, more compounds are assigned excess states, reducing the number of entirely correct and entirely incorrect assignments (Table 2, Additional file 1: Tables S2, S3, and S4). We select *n* = 1.0 as our default, as it strikes a good balance between the three outcomes.

**Table 2:** Dimorphite-DL accuracy

pK_a_ precision factor, *n* (standard deviation)	Correct (%)	Excess (%)	Incorrect (%)
0.0	70.9	23.9	5.2
0.5	69.1	26.5	4.4
1.0	58.8	40.2	0.9
1.5	51.2	48.8	0.0
2.0	50.7	49.3	0.0
2.5	23.9	76.1	0.0
3.0	22.1	77.9	0.0

The percentage of molecules that are correctly/excessively/incorrectly protonated at different pK_a_ precision factors (*n*), at physiological pH (6.4–8.4). To generate these statistics, we considered all 1938 compounds in our primary set, as well as the 78 additional phosphate and phosphonate compounds described in the Additional file 1

Users can specify other values of *n* according to their needs. Tuning the pK_a_ precision factor is particularly useful when one needs to limit the size of the compound library. To illustrate, consider a given library compound with *i* distinct ionizable moieties. Dimorphite-DL will process each moiety separately. If no moiety has a range_PKA_ that overlaps with range_pH_, dimorphite-DL will produce only one protomer. If every range_PKA_ overlaps with range_pH_, dimorphite-DL will produce 2^*i*^ distinct protomers.

Selecting lower values of *n* protects against combinatorial explosions, as range_PKA_ and range_pH_ are less likely to overlap. Narrowing the difference between *pH*_*min*_ and *pH*_*max*_ can further reduce overlap. These measures limit the size of the resulting compound library, reducing the computational cost of any subsequent VS. But restrictive parameters may force dimorphite-DL to ignore binding-relevant states, reducing VS accuracy. Table 2, Additional file 1: Tables S2, S3, and S4 will help the user find a good balance between accuracy, generalizability, and performance.

### Accuracy per ionizable moiety

Next, we evaluated how accurately dimorphite-DL predicts the ionization states of individual moieties (Table 3). To simplify analysis, we considered only *n* = 1.0 and physiological pH (range_pH_ = [6.4, 8.4]). Here, we focus on the amine (1°, 2°, and 3°), carboxylic acid, phenol, benzoic acid, and sulfonamide moieties because they are drug like and are well represented in our 1938-member compound set (21%, 20%, 10%, 7%, and 2%, respectively). A similar analysis of the remaining moieties can be found in the Additional file 1: Tables S2, S3, and S4.

**Table 3:** Dimorphite-DL accuracy at physiological pH (6.4–8.4) for five common moieties

	Correct (%)	Excess (%)	Incorrect (%)
Amine (1°, 2°, and 3°)	26.9 ± 3.0	73.1 ± 3.0	0.0 ± 0.0
Carboxylic acid	100.0 ± 0.0	0.0 ± 0.0	0.0 ± 0.0
Phenol	33.7 ± 3.8	66.3 ± 3.8	0.0 ± 0.0
Benzoic acid	100.0 ± 0.0	0.0 ± 0.0	0.0 ± 0.0
Sulfonamide	37.1 ± 11.6	62.9 ± 11.6	0.0 ± 0.0

Mean ± standard-deviation percentages were calculated using three-fold cross validation. The pK_a_ precision factor (*n*) is 1.0. Additional file 1: Tables S2, S3, and S4 report similar accuracy measures for additional moieties, range_pH_, and *n*

We evaluated each moiety using threefold cross validation. For each fold, we divided all the relevant molecules from our compound set into a training set (two thirds of all samples) and a testing set (the remaining one third of all samples). We calculated the pK_a_ mean (µ) and standard deviation (σ) of the compounds in the training set and defined range_PKA_ to be [µ − 1.0σ, µ + 1.0σ]. To evaluate accuracy, we calculated the percentage of testing-set compounds with correct, excess, and incorrect predicted states. This cross-validation approach was used only to evaluate our model. The published program uses range_PKA_ values derived from all molecules.

### Comparing dimorphite-DL to similar commercial programs

Several other programs can predict small-molecule ionization states. A complete review of these programs is beyond the scope of this work. We direct interested readers to refs. [5, 18–20]. But we do wish to mention a few advantages that dimorphite-DL has over other packages.

Dimorphite-DL is free and open source. Similar commercial programs can be expensive (e.g., Schrödinger’s Epik [1, 4] and Jaguar; BIOVIA’s Pipeline Pilot; etc.). Not all academic researchers can afford the subscription fees, and labs that focus primarily on experimental work cannot justify so large a computational investment.

Some programs (e.g., software by ChemAxon and OpenEye) have “free” academic licenses with concerning commercialization and intellectual-property (IP) restrictions. For example, OpenEye’s free license requires researchers to give up any IP rights and to promptly release their work to the public domain. Eligibility is also regularly reevaluated, and access may be unexpectedly and suddenly withdrawn. Many researchers are reluctant to incorporate commercial tools into existing pipelines, as they limit dissemination.

Dimorphite-DL is well suited for preparing large compound libraries for use in VS. Unlike some other programs (e.g., ChemAxon’s Marvin [5]), dimorphite-DL can process small molecules in batch. Its empirical approach also prepares large libraries quickly. The expensive quantum mechanical calculations used by some other programs (e.g., Schrödinger’s Jaguar) cannot be easily applied at scale. ARChem’s SPARC program, though capable of batch processing, also reportedly suffers from long runtimes [5].

### Comparing dimorphite-DL to Open Babel

The popular cheminformatics program Open Babel [21] is arguably most similar to dimorphite-DL in terms of its license and features. Like dimorphite-DL, Open Babel allows users to ionize small-molecule models as appropriate for a given pH. For each input molecule, Open Babel produces a single output molecule. In contrast, dimorphite-DL can produce multiple outputs, each with different ionization states. Open Babel and dimorphite-DL are also similar in that both are released under open-source licenses. But Open Babel is licensed under the GNU General Public License, a so-called viral license. Many who wish to incorporate an ionization module into their existing software will find this license unacceptable. In contrast, dimorphite-DL is released under the more permissive Apache License, Version 2.0.

Open Babel ionizes small molecules per a rule-based approach similar to that of dimorphite-DL. Excluding its substructure rules specific to amino acids, Open Babel considers 14 generally applicable ionizable substructures. In contrast, dimorphite-DL considers 38 such substructures. There is a one-to-one mapping between many Open-Babel and dimorphite-DL substructure rules. Both programs recognize the same amines, hydroxamic acids, phosphate diesters, phosphonate esters, sulfinic acids, guanidines/amidines, and azides. In other cases, a single Open-Babel substructure maps to multiple dimorphite-DL substructures. For example, unlike Open Babel, dimorphite-DL distinguishes between carboxylates and phenyl carboxylates; phosphates and phosphonates; sulfates and sulfonates; and vinyl alcohols with and without conjugated ketones. Open Babel and dimorphite-DL also handle aromatic nitrogen atoms differently. Whereas Open Babel considers imidazoles and tetrazoles specifically, dimorphite-DL takes a more generalizable approach. When predicting ionization, dimorphite-DL considers only whether an uncharged aromatic nitrogen atom is protonated (e.g., 1*H*-pyrrole) or unprotonated (e.g., pyridine).

Dimorphite-DL also better accounts for phosphate and phosphonate groups. These groups can exist in three ionization states (doubly deprotonated, singly deprotonated, and fully protonated). Dimorphite-DL can generate all three forms, but Open Babel generates only one of two (doubly deprotonated or fully protonated). Dimorphite-DL also generates both protonated and deprotonated azides (pK_a_ = 4.65 [22]); in contrast, Open Babel always protonates azides.

## Limitations

As mentioned above, Dimorphite-DL uses an empirical approach with the advantages of speed and reasonable accuracy, but it does assign ionization states without regard for the larger intramolecular context. To understand why intramolecular effects are at times important, consider phenol (pK_a_ of 9.99 at 25° in water [23]). Adding hydrocarbon substituents to the phenyl ring increases the hydroxyl pK_a_ (e.g., *m*-cresol, 4-(*tert*-butyl)phenol, and 2,6-di-*tert*-butyl-4-methyl-phenol have pK_a_ values of 10.09 [24], 10.32 [25], and 12.55 [26], respectively). In contrast, halide substituents tend to decrease the hydroxyl pK_a_ (e.g., 2,3,4,5,6-pentachlorophenol, 2,4,6-trichlorophenol, 2,4-dichlorophenol, and 4-chlorophenol have pK_a_ values of 4.79 [27], 6.15 [28], 7.85 [29], and 9.59 [25], respectively). Dimorphite-DL considers only the phenol substructure when predicting ionization states. It knows only that phenols in all their forms tend to have hydroxyl pK_a_ values that center around 7.07, with a standard deviation of 3.28 (Table 1).

The same limitation applies to additional moieties that are themselves ionizable. For example, when a second ionizable hydroxyl group is added to a phenol aromatic ring, the pK_a_ is slightly reduced (e.g., pyrocatechol, resorcinol, and hydroquinone have pK_a_ values of 9.25 [30], 9.44 [31], and 9.85 [32], respectively). Ionizable carboxylate groups also impact the pK_a_ (e.g., 4-hydroxybenzoic acid and salicylic acid have hydroxyl pK_a_ values of 9.23 [33] and 13.3 [34], respectively), as do ionizable sulfonate groups (e.g., 4-hydroxybenzenesulfonic acid and 3-hydroxybenzenesulfonic acid have hydroxyl pK_a_ values of 8.7 and 9.07 [35], respectively).

Applying dimorphite-DL to salicylic acid (i.e., 2-hydroxybenzoic acid) at physiological pH (pH 6.4–8.4, default settings) illustrates the occasional pitfalls of our limited-substructure approach. Dimorphite-DL correctly recognized that protonated carboxyl groups are rare at this pH. But it incorrectly predicted that the hydroxyl group exists in both protonated and unprotonated forms. In reality, the pK_a_ of the salicylic acid hydroxyl group is unusually high (13.3 [34]), such that only the protonated hydroxyl is truly prevalent. While salicylic acid presents an admittedly extraordinary use case, we nevertheless welcome future high-throughput methods that take a more whole-molecule approach to ionization-state prediction.

We note also that dimorphite-DL computes ionization states, but not prototropic tautomerization states [36]. To clarify, ionization involves the gain or loss of a proton. Prototropic tautomerization involves intramolecular proton transfer from one atom to another. Existing open-source tools (e.g., MolVS [37]) are available for modeling prototropic tautomerization. Using dimorphite-DL with these other programs will allow researchers to fully enumerate all protonation (i.e., ionization and tautomeric) states.

These limitations aside, we expect that dimorphite-DL will be a useful tool for researchers engaged in structure-based VS. This free and open-source program for predicting small-molecule ionization states will improve VS accuracy, helping to identify novel bioactive molecules.

## Additional file

**Additional file 1.** Supplementary discussion and tables.
