# Supplementary material for: Dimorphite-DL: an open-source program for enumerating the ionization states of drug-like small molecules
Source: J Cheminform. 2019 Feb 14;11:14. doi: 10.1186/s13321-019-0336-9 (PMC6689865; doi:10.1186/s13321-019-0336-9)
Supplement: Supplementary file 1 — Additional file 1. Supplementary discussion and tables. [file 13321_2019_336_MOESM1_ESM.docx]

# Supporting Information

## Phosphates and Phosphonates

The main text describes our work studying ionizable groups that gain or lose a single proton. Of the 38 ionizable groups that dimorphite-DL considers, 36 fall in this category. We determined a single range_PKA_ for each of these 36 moieties by drawing on a set of 1,938 small molecules with experimental pK_a_ values, as described in the Implementation section.

Two of the 38 groups, phosphates and phosphonates, can lose up to two protons. To predict the ionization states of these groups, we separately considered a set of 78 phosphate and phosphonate compounds with experimental pK_a_ values. Both compound classes have two pK_a_ values, pK_a_^1^ and pK_a_^2^. Though the two ionizable oxygen atoms of phosphates and phosphonates are equivalent, let us call one the “first site” (associated with pK_a_^1^) and the other the “second site” (associated with pK_a_^2^). We calculated two pK_a_ means and standard deviations for each site (µ_1_ and σ_1_; and µ_2_ and σ_2_). For phosphates and phosphonates, dimorphite-DL applies the same scheme described above, except it compares two range_PKA_ ranges to range_pH_ rather than just one.

To evaluate how accurately dimorphite-DL predicts the ionization states of phosphates and phosphonates, we used the same correct, excess, and incorrect categories used to assess monoprotic compounds. Applying dimorphite-DL to a phosphate or phosphonate can again have one of three outcomes:

1. Dimorphite-DL is “correct” if it predicts the correct states for both pK_a_^1^ and pK_a_^2^, with no additional states. One of these scenarios must be satisfied:
   1. pK_a_^1^ < *pH_min_*, and dimorphite-DL deprotonates the first site
      1. pK_a_^2^ < *pH_min_*, and dimorphite-DL deprotonates the second site
      2. pK_a_^2^ > *pH_max_*, and dimorphite-DL protonates the second site
      3. *pH_min_* ≤ pK_a_^2^ ≤ *pH_max_*, and dimorphite-DL generates both deprotonated and protonated forms of the second site
   2. pK_a_^1^ > *pH_max_*, and dimorphite-DL protonates the first site
      1. pK_a_^2^ < *pH_min_*, and dimorphite-DL deprotonates the second site
      2. pK_a_^2^ > *pH_max_*, and dimorphite-DL protonates the second site
      3. *pH_min_* ≤ pK_a_^2^ ≤ *pH_max_*, and dimorphite-DL generates both deprotonated and protonated forms of the second site
   3. *pH_min_* ≤ pK_a_^1^ ≤ *pH_max_*, and dimorphite-DL generates both deprotonated and protonated forms of the first site
      1. pK_a_^2^ < *pH_min_*, and dimorphite-DL deprotonates the second site
      2. pK_a_^2^ > *pH_max_*, and dimorphite-DL protonates the second site
      3. *pH_min_* ≤ pK_a_^2^ ≤ *pH_max_*, and dimorphite-DL generates both deprotonated and protonated forms of the second site
2. Dimorphite-DL predicts an “excess” state if, aside from predicting the correct state for both pK_a_^1^ and pK_a_^2^, it also predicts additional states. One of these scenarios must be satisfied:
   1. pK_a_^1^ < *pH_min_*, and dimorphite-DL deprotonates the first site
      1. pK_a_^2^ < *pH_min_* or pK_a_^2^ > *pH_max_*, but dimorphite-DL generates both deprotonated and protonated forms of the second site
   2. pK_a_^1^ > *pH_max_*, and dimorphite-DL protonates the first site
      1. pK_a_^2^ < *pH_min_* or pK_a_^2^ > *pH_max_*, but dimorphite-DL generates both deprotonated and protonated forms of the second site
   3. *pH_min_* ≤ pK_a_^1^ ≤ *pH_max_*, and dimorphite-DL generates both deprotonated and protonated forms of the first site
      1. pK_a_^2^ < *pH_min_* or pK_a_^2^ > *pH_max_*, but dimorphite-DL generates both deprotonated and protonated forms of the second site
   4. pK_a_^1^ < *pH_min_* or pK_a_^1^ > *pH_max_*, but dimorphite-DL generates both deprotonated and protonated forms of the first site
      1. pK_a_^2^ < *pH_min_*, and dimorphite-DL deprotonates the second site
      2. pK_a_^2^ > *pH_max_*, and dimorphite-DL protonates the second site
      3. *pH_min_* ≤ pK_a_^2^ ≤ *pH_max_*, and dimorphite-DL generates both deprotonated and protonated forms of the second site
      4. pK_a_^2^ < *pH_min_* or pK_a_^2^ > *pH_max_*, but dimorphite-DL generates both deprotonated and protonated forms of the second site
3. Dimorphite-DL is “incorrect” (or “incomplete”) if it fails to predict the correct state for pK_a_^1^ or pK_a_^2^ (or both). One of these scenarios must be satisfied:
   1. pK_a_^1^ < *pH_min_*, but dimorphite-DL protonates the first site
   2. pK_a_^1^ > *pH_max_*, but dimorphite-DL deprotonates the first site
   3. *pH_min_* ≤ pK_a_^1^ ≤ *pH_max_*, but dimorphite-DL either deprotonates or protonates the first site (not both)
   4. pK_a_^2^ < *pH_min_*, but dimorphite-DL protonates the second site
   5. pK_a_^2^ > *pH_max_*, but dimorphite-DL deprotonates the second site
   6. *pH_min_* ≤ pK_a_^2^ ≤ *pH_max_*, but dimorphite-DL either deprotonates or protonates the second site (not both)

**Table S1. The 38 ionizable dimorphite-DL substructures.** Substructures are listed in order of decreasing priority. The Atom Index indicates the SMARTS atom that can be protonated or deprotonated.

| **Substructure ID** | SMARTS | Index |
| --- | --- | --- |
| Azide | [N+0:1]=[N+:2]=[N+0:3]-[H] | 2 |
| Nitro | [C,c,N,n,O,o:1]-[NX3:2](=[O:3])-[O:4]-[H] | 3 |
| AmidineGuanidine1 | [N:1]-[C:2](-[N:3])=[NX2:4]-[H:5] | 3 |
| AmidineGuanidine2 | [C:1](-[N:2])=[NX2+0:3]-[H] | 2 |
| Sulfate | [SX4:1](=[O:2])(=[O:3])([O:4]-[C,c,N,n:5])-[OX2:6]-[H] | 5 |
| Sulfonate | [SX4:1](=[O:2])(=[O:3])(-[C,c,N,n:4])-[OX2:5]-[H] | 4 |
| Sulfinic Acid | [SX3:1](=[O:2])-[O:3]-[H] | 2 |
| Phenyl_carboxyl | [c,n,o:1]-[C:2](=[O:3])-[O:4]-[H] | 3 |
| Carboxyl | [C:1](=[O:2])-[O:3]-[H] | 2 |
| Thioic Acid | [C,c,N,n:1](=[O,S:2])-[SX2,OX2:3]-[H] | 2 |
| Phenyl_Thiol | [c,n:1]-[SX2:2]-[H] | 1 |
| Thiol | [C,N:1]-[SX2:2]-[H] | 1 |
| Phosphate | [PX4:1](=[O:2])(-[OX2:3]-[H])(-[O+0:4])-[OX2:5]-[H] | 2, 5 |
| Phosphonate | [PX4:1](=[O:2])(-[OX2:3]-[H])(-[C,c,N,n:4])-[OX2:5]-[H] | 2, 5 |
| Phenol | [c,n,o:1]-[O:2]-[H] | 1 |
| Peroxide1 | [O:1]([$(C=O),$(C[Cl]),$(CF),$(C[Br]),$(CC#N):2])-[O:3]-[H] | 2 |
| Peroxide2 | [C:1]-[O:2]-[O:3]-[H] | 2 |
| O=C-C=C-OH | [O:1]=[C;R:2]-[C;R:3]=[C;R:4]-[O:3]-[H] | 4 |
| Vinyl Alcohol | [C:1]=[C:2]-[O:3]-[H] | 2 |
| Alcohol | [C:1]-[O:2]-[H] | 1 |
| N-hydroxyamide | [C:1](=[O:2])-[N:3]-[O:4]-[H] | 3 |
| Ringed_imide1 | [O,S:1]=[C;R:2]([$([#8]),$([#7]),$([#16]),$([#6][Cl]),$([#6]F),$([#6][Br]):3])-[N;R:4]([C;R:5]=[O,S:6])-[H] | 3 |
| Ringed_imide2 | [O,S:1]=[C;R:2]-[N;R:3]([C;R:4]=[O,S:5])-[H] | 2 |
| Imide | [F,Cl,Br,S,s,P,p:1][#6:2][CX3:3](=[O,S:4])-[NX3+0:5]([CX3:6]=[O,S:7])-[H] | 4 |
| Imide2 | [O,S:1]=[CX3:2]-[NX3+0:3]([CX3:4]=[O,S:5])-[H] | 2 |
| *Amide_electronegative | [C:1](=[O:2])-[N:3](-[Br,Cl,I,F,S,O,N,P:4])-[H] | 2 |
| *Amide | [C:1](=[O:2])-[N:3]-[H] | 2 |
| *Sulfonamide | [SX4:1](=[O:2])(=[O:3])-[NX3+0:4]-[H] | 3 |
| Anilines_primary | [c:1]-[NX3+0:2]([H:3])[H:4] | 1 |
| Anilines_secondary | [c:1]-[NX3+0:2]([H:3])[!H:4] | 1 |
| Anilines_tertiary | [c:1]-[NX3+1:2]([!H:3])([!H:4])-[H] | 1 |
| Aromatic_nitrogen_unprotonated | [n+1&H1:1] | 0 |
| Amines_primary_secondary_tertiary | [C:1]-[NX4+1:2]-[H] | 1 |
| Phosphinic Acid | [PX4:1](=[O:2])(-[C,c,N,n,F,Cl,Br,I:3])(-[C,c,N,n,F,Cl,Br,I:4])-[OX2:5]-[H] | 4 |
| Phosphate Diester | [PX4:1](=[O:2])(-[OX2:3]-[C,c,N,n,F,Cl,Br,I:4])(-[O+0:5]-[C,c,N,n,F,Cl,Br,I:4])-[OX2:6]-[H] | 6 |
| Phosphonate Ester | [PX4:1](=[O:2])(-[OX2:3]-[C,c,N,n,F,Cl,Br,I:4])(-[C,c,N,n,F,Cl,Br,I:5])-[OX2:6]-[H] | 5 |
| Primary_hydroxyl_amine | [C,c:1]-[O:2]-[NH2:3] | 2 |
| Aromatic_nitrogen_protonated | [n:1]-[H] | 0 |

**Table S2: Dimorphite-DL accuracy at physiological pH (6.4-8.4), assessed with three-fold cross validation.** The percentage of each moiety with correct, excess, and incorrect predicted ionization states for various pK_a_ precision factors (stdev). Azide and nitro groups were not included in the analysis because there were too few in our compound set to perform three-fold cross validation.

|  |  | stdev: 0.0 | stdev: 1.0 | stdev: 1.5 | stdev: 2.0 |
| --- | --- | --- | --- | --- | --- |
| Alcohol | Correct (%)  Excess (%)  Incorrect (%) | 100.0 ± 0.0  0.0 ± 0.0  0.0 ± 0.0 | 100.0 ± 0.0  0.0 ± 0.0  0.0 ± 0.0 | 100.0 ± 0.0  0.0 ± 0.0  0.0 ± 0.0 | 100.0 ± 0.0  0.0 ± 0.0  0.0 ± 0.0 |
| Amide | Correct (%)  Excess (%)  Incorrect (%) | 83.3 ± 13.6  0.0 ± 0.0  16.7 ± 13.6 | 22.2 ± 31.4  66.7 ± 47.1  11.1 ± 15.7 | 5.6 ± 7.9  94.4 ± 7.9  0.0 ± 0.0 | 5.6 ± 7.9  94.4 ± 7.9  0.0 ± 0.0 |
| Amide_electronegative | Correct (%)  Excess (%)  Incorrect (%) | 84.3 ± 4.6  0.0 ± 0.0  15.7 ± 4.6 | 59.3 ± 33.3  29.2 ± 41.2  11.6 ± 9.1 | 7.9 ± 5.6  92.1 ± 5.6  0.0 ± 0.0 | 7.9 ± 5.6  92.1 ± 5.6  0.0 ± 0.0 |
| AmidineGuanidine1 | Correct (%)  Excess (%)  Incorrect (%) | 93.3 ± 9.4  0.0 ± 0.0  6.7 ± 9.4 | 93.3 ± 9.4  0.0 ± 0.0  6.7 ± 9.4 | 93.3 ± 9.4  0.0 ± 0.0  6.7 ± 9.4 | 60.0 ± 43.2  33.3 ± 47.1  6.7 ± 9.4 |
| AmidineGuanidine2 | Correct (%)  Excess (%)  Incorrect (%) | 75.5 ± 3.9  0.0 ± 0.0  24.5 ± 3.9 | 21.5 ± 4.1  78.5 ± 4.1  0.0 ± 0.0 | 21.5 ± 4.1  78.5 ± 4.1  0.0 ± 0.0 | 21.5 ± 4.1  78.5 ± 4.1  0.0 ± 0.0 |
| Amines_primary_secondary_tertiary | Correct (%)  Excess (%)  Incorrect (%) | 26.9 ± 3.0  73.1 ± 3.0  0.0 ± 0.0 | 26.9 ± 3.0  73.1 ± 3.0  0.0 ± 0.0 | 26.9 ± 3.0  73.1 ± 3.0  0.0 ± 0.0 | 26.9 ± 3.0  73.1 ± 3.0  0.0 ± 0.0 |
| Anilines_primary | Correct (%)  Excess (%)  Incorrect (%) | 94.8 ± 3.7  0.0 ± 0.0  5.2 ± 3.7 | 61.4 ± 43.4  33.3 ± 47.1  5.2 ± 3.7 | 30.7 ± 43.4  66.7 ± 47.1  2.7 ± 3.8 | 0.0 ± 0.0  100.0 ± 0.0  0.0 ± 0.0 |
| Anilines_secondary | Correct (%)  Excess (%)  Incorrect (%) | 83.7 ± 2.7  0.0 ± 0.0  16.3 ± 2.7 | 14.2 ± 2.4  85.8 ± 2.4  0.0 ± 0.0 | 14.2 ± 2.4  85.8 ± 2.4  0.0 ± 0.0 | 14.2 ± 2.4  85.8 ± 2.4  0.0 ± 0.0 |
| Anilines_tertiary | Correct (%)  Excess (%)  Incorrect (%) | 84.2 ± 8.6  0.0 ± 0.0  15.8 ± 8.6 | 84.2 ± 8.6  0.0 ± 0.0  15.8 ± 8.6 | 14.0 ± 6.6  86.0 ± 6.6  0.0 ± 0.0 | 14.0 ± 6.6  86.0 ± 6.6  0.0 ± 0.0 |
| Aromatic_nitrogen_protonated | Correct (%)  Excess (%)  Incorrect (%) | 75.0 ± 10.2  0.0 ± 0.0  25.0 ± 10.2 | 8.3 ± 5.9  91.7 ± 5.9  0.0 ± 0.0 | 8.3 ± 5.9  91.7 ± 5.9  0.0 ± 0.0 | 8.3 ± 5.9  91.7 ± 5.9  0.0 ± 0.0 |
| Aromatic_nitrogen_unprotonated | Correct (%)  Excess (%)  Incorrect (%) | 88.0 ± 2.7  0.0 ± 0.0  12.0 ± 2.7 | 35.1 ± 36.2  60.2 ± 42.7  4.6 ± 6.5 | 9.2 ± 3.5  90.8 ± 3.5  0.0 ± 0.0 | 9.2 ± 3.5  90.8 ± 3.5  0.0 ± 0.0 |
| Carboxyl | Correct (%)  Excess (%)  Incorrect (%) | 100.0 ± 0.0  0.0 ± 0.0  0.0 ± 0.0 | 100.0 ± 0.0  0.0 ± 0.0  0.0 ± 0.0 | 100.0 ± 0.0  0.0 ± 0.0  0.0 ± 0.0 | 100.0 ± 0.0  0.0 ± 0.0  0.0 ± 0.0 |
| Imide | Correct (%)  Excess (%)  Incorrect (%) | 100.0 ± 0.0  0.0 ± 0.0  0.0 ± 0.0 | 100.0 ± 0.0  0.0 ± 0.0  0.0 ± 0.0 | 100.0 ± 0.0  0.0 ± 0.0  0.0 ± 0.0 | 66.7 ± 47.1  33.3 ± 47.1  0.0 ± 0.0 |
| Imide2 | Correct (%)  Excess (%)  Incorrect (%) | 100.0 ± 0.0  0.0 ± 0.0  0.0 ± 0.0 | 100.0 ± 0.0  0.0 ± 0.0  0.0 ± 0.0 | 66.7 ± 47.1  33.3 ± 47.1  0.0 ± 0.0 | 33.3 ± 47.1  66.7 ± 47.1  0.0 ± 0.0 |
| N-hydroxyamide | Correct (%)  Excess (%)  Incorrect (%) | 61.9 ± 33.7  0.0 ± 0.0  38.1 ± 33.7 | 14.3 ± 0.0  57.1 ± 40.4  28.6 ± 40.4 | 38.1 ± 33.7  61.9 ± 33.7  0.0 ± 0.0 | 38.1 ± 33.7  61.9 ± 33.7  0.0 ± 0.0 |
| O=C-C=C-OH | Correct (%)  Excess (%)  Incorrect (%) | 100.0 ± 0.0  0.0 ± 0.0  0.0 ± 0.0 | 100.0 ± 0.0  0.0 ± 0.0  0.0 ± 0.0 | 100.0 ± 0.0  0.0 ± 0.0  0.0 ± 0.0 | 100.0 ± 0.0  0.0 ± 0.0  0.0 ± 0.0 |
| Peroxide1 | Correct (%)  Excess (%)  Incorrect (%) | 72.2 ± 20.8  0.0 ± 0.0  27.8 ± 20.8 | 27.8 ± 20.8  72.2 ± 20.8  0.0 ± 0.0 | 27.8 ± 20.8  72.2 ± 20.8  0.0 ± 0.0 | 27.8 ± 20.8  72.2 ± 20.8  0.0 ± 0.0 |
| Peroxide2 | Correct (%)  Excess (%)  Incorrect (%) | 100.0 ± 0.0  0.0 ± 0.0  0.0 ± 0.0 | 100.0 ± 0.0  0.0 ± 0.0  0.0 ± 0.0 | 100.0 ± 0.0  0.0 ± 0.0  0.0 ± 0.0 | 100.0 ± 0.0  0.0 ± 0.0  0.0 ± 0.0 |
| Phenol | Correct (%)  Excess (%)  Incorrect (%) | 33.7 ± 3.8  66.3 ± 3.8  0.0 ± 0.0 | 33.7 ± 3.8  66.3 ± 3.8  0.0 ± 0.0 | 33.7 ± 3.8  66.3 ± 3.8  0.0 ± 0.0 | 33.7 ± 3.8  66.3 ± 3.8  0.0 ± 0.0 |
| Phenyl_carboxyl | Correct (%)  Excess (%)  Incorrect (%) | 100.0 ± 0.0  0.0 ± 0.0  0.0 ± 0.0 | 100.0 ± 0.0  0.0 ± 0.0  0.0 ± 0.0 | 100.0 ± 0.0  0.0 ± 0.0  0.0 ± 0.0 | 100.0 ± 0.0  0.0 ± 0.0  0.0 ± 0.0 |
| Phenyl_thiol | Correct (%)  Excess (%)  Incorrect (%) | 77.8 ± 15.7  0.0 ± 0.0  22.2 ± 15.7 | 16.7 ± 13.6  83.3 ± 13.6  0.0 ± 0.0 | 16.7 ± 13.6  83.3 ± 13.6  0.0 ± 0.0 | 16.7 ± 13.6  83.3 ± 13.6  0.0 ± 0.0 |
| Phosphate | Correct (%)  Excess (%)  Incorrect (%) | 70.4 ± 5.2  18.5 ± 13.9  11.1 ± 15.7 | 63.0 ± 13.9  37.0 ± 13.9  0.0 ± 0.0 | 63.0 ± 13.9  37.0 ± 13.9  0.0 ± 0.0 | 63.0 ± 13.9  37.0 ± 13.9  0.0 ± 0.0 |
| Phosphate_diester | Correct (%)  Excess (%)  Incorrect (%) | 95.2 ± 6.7  0.0 ± 0.0  4.8 ± 6.7 | 95.2 ± 6.7  0.0 ± 0.0  4.8 ± 6.7 | 28.6 ± 40.4  66.7 ± 47.1  4.8 ± 6.7 | 28.6 ± 40.4  66.7 ± 47.1  4.8 ± 6.7 |
| Phosphinic_acid | Correct (%)  Excess (%)  Incorrect (%) | 100.0 ± 0.0  0.0 ± 0.0  0.0 ± 0.0 | 100.0 ± 0.0  0.0 ± 0.0  0.0 ± 0.0 | 100.0 ± 0.0  0.0 ± 0.0  0.0 ± 0.0 | 100.0 ± 0.0  0.0 ± 0.0  0.0 ± 0.0 |
| Phosphonate | Correct (%)  Excess (%)  Incorrect (%) | 76.5 ± 14.4  23.5 ± 14.4  0.0 ± 0.0 | 76.5 ± 14.4  23.5 ± 14.4  0.0 ± 0.0 | 76.5 ± 14.4  23.5 ± 14.4  0.0 ± 0.0 | 76.5 ± 14.4  23.5 ± 14.4  0.0 ± 0.0 |
| Phosphonate_ester | Correct (%)  Excess (%)  Incorrect (%) | 100.0 ± 0.0  0.0 ± 0.0  0.0 ± 0.0 | 100.0 ± 0.0  0.0 ± 0.0  0.0 ± 0.0 | 100.0 ± 0.0  0.0 ± 0.0  0.0 ± 0.0 | 100.0 ± 0.0  0.0 ± 0.0  0.0 ± 0.0 |
| Primary_hydroxyl_amine | Correct (%)  Excess (%)  Incorrect (%) | 100.0 ± 0.0  0.0 ± 0.0  0.0 ± 0.0 | 100.0 ± 0.0  0.0 ± 0.0  0.0 ± 0.0 | 100.0 ± 0.0  0.0 ± 0.0  0.0 ± 0.0 | 100.0 ± 0.0  0.0 ± 0.0  0.0 ± 0.0 |
| Ringed_imide1 | Correct (%)  Excess (%)  Incorrect (%) | 11.1 ± 15.7  33.3 ± 47.1  55.6 ± 41.6 | 55.6 ± 41.6  44.4 ± 41.6  0.0 ± 0.0 | 55.6 ± 41.6  44.4 ± 41.6  0.0 ± 0.0 | 55.6 ± 41.6  44.4 ± 41.6  0.0 ± 0.0 |
| Ringed_imide2 | Correct (%)  Excess (%)  Incorrect (%) | 58.3 ± 21.2  20.8 ± 29.5  20.8 ± 21.2 | 12.5 ± 17.7  87.5 ± 17.7  0.0 ± 0.0 | 12.5 ± 17.7  87.5 ± 17.7  0.0 ± 0.0 | 12.5 ± 17.7  87.5 ± 17.7  0.0 ± 0.0 |
| Sulfate | Correct (%)  Excess (%)  Incorrect (%) | 100.0 ± 0.0  0.0 ± 0.0  0.0 ± 0.0 | 100.0 ± 0.0  0.0 ± 0.0  0.0 ± 0.0 | 100.0 ± 0.0  0.0 ± 0.0  0.0 ± 0.0 | 100.0 ± 0.0  0.0 ± 0.0  0.0 ± 0.0 |
| Sulfinic_acid | Correct (%)  Excess (%)  Incorrect (%) | 100.0 ± 0.0  0.0 ± 0.0  0.0 ± 0.0 | 100.0 ± 0.0  0.0 ± 0.0  0.0 ± 0.0 | 100.0 ± 0.0  0.0 ± 0.0  0.0 ± 0.0 | 100.0 ± 0.0  0.0 ± 0.0  0.0 ± 0.0 |
| Sulfonamide | Correct (%)  Excess (%)  Incorrect (%) | 37.1 ± 11.6  62.9 ± 11.6  0.0 ± 0.0 | 37.1 ± 11.6  62.9 ± 11.6  0.0 ± 0.0 | 37.1 ± 11.6  62.9 ± 11.6  0.0 ± 0.0 | 37.1 ± 11.6  62.9 ± 11.6  0.0 ± 0.0 |
| Sulfonate | Correct (%)  Excess (%)  Incorrect (%) | 100.0 ± 0.0  0.0 ± 0.0  0.0 ± 0.0 | 100.0 ± 0.0  0.0 ± 0.0  0.0 ± 0.0 | 100.0 ± 0.0  0.0 ± 0.0  0.0 ± 0.0 | 100.0 ± 0.0  0.0 ± 0.0  0.0 ± 0.0 |
| Thioic_acid | Correct (%)  Excess (%)  Incorrect (%) | 100.0 ± 0.0  0.0 ± 0.0  0.0 ± 0.0 | 100.0 ± 0.0  0.0 ± 0.0  0.0 ± 0.0 | 100.0 ± 0.0  0.0 ± 0.0  0.0 ± 0.0 | 100.0 ± 0.0  0.0 ± 0.0  0.0 ± 0.0 |
| Thiol | Correct (%)  Excess (%)  Incorrect (%) | 61.9 ± 6.0  0.0 ± 0.0  38.1 ± 6.0 | 38.1 ± 6.0  61.9 ± 6.0  0.0 ± 0.0 | 38.1 ± 6.0  61.9 ± 6.0  0.0 ± 0.0 | 38.1 ± 6.0  61.9 ± 6.0  0.0 ± 0.0 |
| Vinyl_alcohol | Correct (%)  Excess (%)  Incorrect (%) | 81.0 ± 6.7  0.0 ± 0.0  19.0 ± 6.7 | 9.5 ± 6.7  90.5 ± 6.7  0.0 ± 0.0 | 9.5 ± 6.7  90.5 ± 6.7  0.0 ± 0.0 | 9.5 ± 6.7  90.5 ± 6.7  0.0 ± 0.0 |
| Average | Correct (%)  Excess (%)  Incorrect (%) | 81.3  8.3  10.4 | 64.1  33.5  2.5 | 56.5  43.1  0.4 | 52.9  46.8  0.3 |
|  |  |  |  |  |  |
|  |  |  |  |  |  |

**Table S3: Dimorphite-DL accuracy at lysosomal pH (4.5-5.0), assessed with three-fold cross validation.** The percentage of each moiety with correct, excess, and incorrect predicted ionization states for various pK_a_ precision factors (stdev).

|  |  | stdev: 0.0 | stdev: 1.0 | stdev: 1.5 | stdev: 2.0 |
| --- | --- | --- | --- | --- | --- |
| Alcohol | Correct (%)  Excess (%)  Incorrect (%) | 100.0 ± 0.0  0.0 ± 0.0  0.0 ± 0.0 | 100.0 ± 0.0  0.0 ± 0.0  0.0 ± 0.0 | 100.0 ± 0.0  0.0 ± 0.0  0.0 ± 0.0 | 100.0 ± 0.0  0.0 ± 0.0  0.0 ± 0.0 |
| Amide | Correct (%)  Excess (%)  Incorrect (%) | 88.9 ± 7.9  0.0 ± 0.0  11.1 ± 7.9 | 88.9 ± 7.9  0.0 ± 0.0  11.1 ± 7.9 | 55.6 ± 39.3  33.3 ± 47.1  11.1 ± 7.9 | 0.0 ± 0.0  100.0 ± 0.0  0.0 ± 0.0 |
| Amide_electronegative | Correct (%)  Excess (%)  Incorrect (%) | 64.4 ± 8.0  0.0 ± 0.0  35.6 ± 8.0 | 12.0 ± 0.7  88.0 ± 0.7  0.0 ± 0.0 | 12.0 ± 0.7  88.0 ± 0.7  0.0 ± 0.0 | 12.0 ± 0.7  88.0 ± 0.7  0.0 ± 0.0 |
| AmidineGuanidine1 | Correct (%)  Excess (%)  Incorrect (%) | 100.0 ± 0.0  0.0 ± 0.0  0.0 ± 0.0 | 100.0 ± 0.0  0.0 ± 0.0  0.0 ± 0.0 | 100.0 ± 0.0  0.0 ± 0.0  0.0 ± 0.0 | 100.0 ± 0.0  0.0 ± 0.0  0.0 ± 0.0 |
| AmidineGuanidine2 | Correct (%)  Excess (%)  Incorrect (%) | 97.0 ± 4.3  0.0 ± 0.0  3.0 ± 4.3 | 97.0 ± 4.3  0.0 ± 0.0  3.0 ± 4.3 | 97.0 ± 4.3  0.0 ± 0.0  3.0 ± 4.3 | 97.0 ± 4.3  0.0 ± 0.0  3.0 ± 4.3 |
| Amines_primary_secondary_tertiary | Correct (%)  Excess (%)  Incorrect (%) | 90.8 ± 0.9  0.0 ± 0.0  9.2 ± 0.9 | 90.8 ± 0.9  0.0 ± 0.0  9.2 ± 0.9 | 2.2 ± 0.0  97.8 ± 0.0  0.0 ± 0.0 | 2.2 ± 0.0  97.8 ± 0.0  0.0 ± 0.0 |
| Anilines_primary | Correct (%)  Excess (%)  Incorrect (%) | 73.9 ± 4.2  0.0 ± 0.0  26.1 ± 4.2 | 15.6 ± 0.3  84.4 ± 0.3  0.0 ± 0.0 | 15.6 ± 0.3  84.4 ± 0.3  0.0 ± 0.0 | 15.6 ± 0.3  84.4 ± 0.3  0.0 ± 0.0 |
| Anilines_secondary | Correct (%)  Excess (%)  Incorrect (%) | 45.0 ± 8.1  0.0 ± 0.0  55.0 ± 8.1 | 16.3 ± 7.7  83.7 ± 7.7  0.0 ± 0.0 | 16.3 ± 7.7  83.7 ± 7.7  0.0 ± 0.0 | 16.3 ± 7.7  83.7 ± 7.7  0.0 ± 0.0 |
| Anilines_tertiary | Correct (%)  Excess (%)  Incorrect (%) | 57.9 ± 4.3  0.0 ± 0.0  42.1 ± 4.3 | 10.5 ± 4.3  89.5 ± 4.3  0.0 ± 0.0 | 10.5 ± 4.3  89.5 ± 4.3  0.0 ± 0.0 | 10.5 ± 4.3  89.5 ± 4.3  0.0 ± 0.0 |
| Aromatic_nitrogen_protonated | Correct (%)  Excess (%)  Incorrect (%) | 87.5 ± 0.0  0.0 ± 0.0  12.5 ± 0.0 | 87.5 ± 0.0  0.0 ± 0.0  12.5 ± 0.0 | 0.0 ± 0.0  100.0 ± 0.0  0.0 ± 0.0 | 0.0 ± 0.0  100.0 ± 0.0  0.0 ± 0.0 |
| Aromatic_nitrogen_unprotonated | Correct (%)  Excess (%)  Incorrect (%) | 34.2 ± 19.7  30.6 ± 43.3  35.2 ± 25.8 | 13.8 ± 4.0  86.2 ± 4.0  0.0 ± 0.0 | 13.8 ± 4.0  86.2 ± 4.0  0.0 ± 0.0 | 13.8 ± 4.0  86.2 ± 4.0  0.0 ± 0.0 |
| Carboxyl | Correct (%)  Excess (%)  Incorrect (%) | 75.7 ± 1.4  0.0 ± 0.0  24.3 ± 1.4 | 19.9 ± 1.8  80.1 ± 1.8  0.0 ± 0.0 | 19.9 ± 1.8  80.1 ± 1.8  0.0 ± 0.0 | 19.9 ± 1.8  80.1 ± 1.8  0.0 ± 0.0 |
| Imide | Correct (%)  Excess (%)  Incorrect (%) | 100.0 ± 0.0  0.0 ± 0.0  0.0 ± 0.0 | 33.3 ± 47.1  66.7 ± 47.1  0.0 ± 0.0 | 33.3 ± 47.1  66.7 ± 47.1  0.0 ± 0.0 | 33.3 ± 47.1  66.7 ± 47.1  0.0 ± 0.0 |
| Imide2 | Correct (%)  Excess (%)  Incorrect (%) | 100.0 ± 0.0  0.0 ± 0.0  0.0 ± 0.0 | 100.0 ± 0.0  0.0 ± 0.0  0.0 ± 0.0 | 100.0 ± 0.0  0.0 ± 0.0  0.0 ± 0.0 | 100.0 ± 0.0  0.0 ± 0.0  0.0 ± 0.0 |
| N-hydroxyamide | Correct (%)  Excess (%)  Incorrect (%) | 100.0 ± 0.0  0.0 ± 0.0  0.0 ± 0.0 | 100.0 ± 0.0  0.0 ± 0.0  0.0 ± 0.0 | 100.0 ± 0.0  0.0 ± 0.0  0.0 ± 0.0 | 100.0 ± 0.0  0.0 ± 0.0  0.0 ± 0.0 |
| O=C-C=C-OH | Correct (%)  Excess (%)  Incorrect (%) | 93.3 ± 9.4  0.0 ± 0.0  6.7 ± 9.4 | 93.3 ± 9.4  0.0 ± 0.0  6.7 ± 9.4 | 0.0 ± 0.0  100.0 ± 0.0  0.0 ± 0.0 | 0.0 ± 0.0  100.0 ± 0.0  0.0 ± 0.0 |
| Peroxide1 | Correct (%)  Excess (%)  Incorrect (%) | 100.0 ± 0.0  0.0 ± 0.0  0.0 ± 0.0 | 100.0 ± 0.0  0.0 ± 0.0  0.0 ± 0.0 | 100.0 ± 0.0  0.0 ± 0.0  0.0 ± 0.0 | 100.0 ± 0.0  0.0 ± 0.0  0.0 ± 0.0 |
| Peroxide2 | Correct (%)  Excess (%)  Incorrect (%) | 100.0 ± 0.0  0.0 ± 0.0  0.0 ± 0.0 | 100.0 ± 0.0  0.0 ± 0.0  0.0 ± 0.0 | 100.0 ± 0.0  0.0 ± 0.0  0.0 ± 0.0 | 100.0 ± 0.0  0.0 ± 0.0  0.0 ± 0.0 |
| Phenol | Correct (%)  Excess (%)  Incorrect (%) | 83.2 ± 2.7  0.0 ± 0.0  16.8 ± 2.7 | 0.5 ± 0.7  99.5 ± 0.7  0.0 ± 0.0 | 0.5 ± 0.7  99.5 ± 0.7  0.0 ± 0.0 | 0.5 ± 0.7  99.5 ± 0.7  0.0 ± 0.0 |
| Phenyl_carboxyl | Correct (%)  Excess (%)  Incorrect (%) | 92.2 ± 3.6  0.0 ± 0.0  7.8 ± 3.6 | 2.8 ± 2.7  97.2 ± 2.7  0.0 ± 0.0 | 2.8 ± 2.7  97.2 ± 2.7  0.0 ± 0.0 | 2.8 ± 2.7  97.2 ± 2.7  0.0 ± 0.0 |
| Phenyl_thiol | Correct (%)  Excess (%)  Incorrect (%) | 42.2 ± 30.0  0.0 ± 0.0  57.8 ± 30.0 | 5.6 ± 7.9  94.4 ± 7.9  0.0 ± 0.0 | 5.6 ± 7.9  94.4 ± 7.9  0.0 ± 0.0 | 5.6 ± 7.9  94.4 ± 7.9  0.0 ± 0.0 |
| Phosphate | Correct (%)  Excess (%)  Incorrect (%) | 92.6 ± 5.2  0.0 ± 0.0  7.4 ± 5.2 | 92.6 ± 5.2  0.0 ± 0.0  7.4 ± 5.2 | 66.7 ± 31.4  25.9 ± 36.7  7.4 ± 5.2 | 33.3 ± 39.5  63.0 ± 44.8  3.7 ± 5.2 |
| Phosphate_diester | Correct (%)  Excess (%)  Incorrect (%) | 95.2 ± 6.7  0.0 ± 0.0  4.8 ± 6.7 | 28.6 ± 40.4  66.7 ± 47.1  4.8 ± 6.7 | 28.6 ± 40.4  66.7 ± 47.1  4.8 ± 6.7 | 0.0 ± 0.0  100.0 ± 0.0  0.0 ± 0.0 |
| Phosphinic_acid | Correct (%)  Excess (%)  Incorrect (%) | 100.0 ± 0.0  0.0 ± 0.0  0.0 ± 0.0 | 100.0 ± 0.0  0.0 ± 0.0  0.0 ± 0.0 | 100.0 ± 0.0  0.0 ± 0.0  0.0 ± 0.0 | 33.3 ± 47.1  66.7 ± 47.1  0.0 ± 0.0 |
| Phosphonate | Correct (%)  Excess (%)  Incorrect (%) | 98.0 ± 2.8  0.0 ± 0.0  2.0 ± 2.8 | 98.0 ± 2.8  0.0 ± 0.0  2.0 ± 2.8 | 98.0 ± 2.8  0.0 ± 0.0  2.0 ± 2.8 | 98.0 ± 2.8  0.0 ± 0.0  2.0 ± 2.8 |
| Phosphonate_ester | Correct (%)  Excess (%)  Incorrect (%) | 100.0 ± 0.0  0.0 ± 0.0  0.0 ± 0.0 | 100.0 ± 0.0  0.0 ± 0.0  0.0 ± 0.0 | 100.0 ± 0.0  0.0 ± 0.0  0.0 ± 0.0 | 100.0 ± 0.0  0.0 ± 0.0  0.0 ± 0.0 |
| Primary_hydroxyl_amine | Correct (%)  Excess (%)  Incorrect (%) | 83.3 ± 23.6  0.0 ± 0.0  16.7 ± 23.6 | 16.7 ± 23.6  83.3 ± 23.6  0.0 ± 0.0 | 16.7 ± 23.6  83.3 ± 23.6  0.0 ± 0.0 | 16.7 ± 23.6  83.3 ± 23.6  0.0 ± 0.0 |
| Ringed_imide1 | Correct (%)  Excess (%)  Incorrect (%) | 100.0 ± 0.0  0.0 ± 0.0  0.0 ± 0.0 | 100.0 ± 0.0  0.0 ± 0.0  0.0 ± 0.0 | 100.0 ± 0.0  0.0 ± 0.0  0.0 ± 0.0 | 100.0 ± 0.0  0.0 ± 0.0  0.0 ± 0.0 |
| Ringed_imide2 | Correct (%)  Excess (%)  Incorrect (%) | 95.8 ± 5.9  0.0 ± 0.0  4.2 ± 5.9 | 95.8 ± 5.9  0.0 ± 0.0  4.2 ± 5.9 | 95.8 ± 5.9  0.0 ± 0.0  4.2 ± 5.9 | 29.2 ± 41.2  66.7 ± 47.1  4.2 ± 5.9 |
| Sulfate | Correct (%)  Excess (%)  Incorrect (%) | 100.0 ± 0.0  0.0 ± 0.0  0.0 ± 0.0 | 100.0 ± 0.0  0.0 ± 0.0  0.0 ± 0.0 | 100.0 ± 0.0  0.0 ± 0.0  0.0 ± 0.0 | 100.0 ± 0.0  0.0 ± 0.0  0.0 ± 0.0 |
| Sulfinic_acid | Correct (%)  Excess (%)  Incorrect (%) | 100.0 ± 0.0  0.0 ± 0.0  0.0 ± 0.0 | 100.0 ± 0.0  0.0 ± 0.0  0.0 ± 0.0 | 100.0 ± 0.0  0.0 ± 0.0  0.0 ± 0.0 | 100.0 ± 0.0  0.0 ± 0.0  0.0 ± 0.0 |
| Sulfonamide | Correct (%)  Excess (%)  Incorrect (%) | 87.1 ± 4.9  0.0 ± 0.0  12.9 ± 4.9 | 87.1 ± 4.9  0.0 ± 0.0  12.9 ± 4.9 | 58.2 ± 36.5  31.1 ± 44.0  10.7 ± 7.9 | 4.3 ± 3.0  95.7 ± 3.0  0.0 ± 0.0 |
| Sulfonate | Correct (%)  Excess (%)  Incorrect (%) | 100.0 ± 0.0  0.0 ± 0.0  0.0 ± 0.0 | 100.0 ± 0.0  0.0 ± 0.0  0.0 ± 0.0 | 100.0 ± 0.0  0.0 ± 0.0  0.0 ± 0.0 | 100.0 ± 0.0  0.0 ± 0.0  0.0 ± 0.0 |
| Thioic_acid | Correct (%)  Excess (%)  Incorrect (%) | 100.0 ± 0.0  0.0 ± 0.0  0.0 ± 0.0 | 100.0 ± 0.0  0.0 ± 0.0  0.0 ± 0.0 | 100.0 ± 0.0  0.0 ± 0.0  0.0 ± 0.0 | 100.0 ± 0.0  0.0 ± 0.0  0.0 ± 0.0 |
| Thiol | Correct (%)  Excess (%)  Incorrect (%) | 100.0 ± 0.0  0.0 ± 0.0  0.0 ± 0.0 | 100.0 ± 0.0  0.0 ± 0.0  0.0 ± 0.0 | 100.0 ± 0.0  0.0 ± 0.0  0.0 ± 0.0 | 100.0 ± 0.0  0.0 ± 0.0  0.0 ± 0.0 |
| Vinyl_alcohol | Correct (%)  Excess (%)  Incorrect (%) | 95.2 ± 6.7  0.0 ± 0.0  4.8 ± 6.7 | 95.2 ± 6.7  0.0 ± 0.0  4.8 ± 6.7 | 95.2 ± 6.7  0.0 ± 0.0  4.8 ± 6.7 | 61.9 ± 44.2  33.3 ± 47.1  4.8 ± 6.7 |
| Average | Correct (%)  Excess (%)  Incorrect (%) | 88.2  0.9  11.0 | 69.5  28.3  2.2 | 59.6  39.1  1.3 | 50.2  49.3  0.5 |
|  |  |  |  |  |  |

**Table S4: Dimorphite-DL accuracy at stomach pH (1.5-3.5), assessed with three-fold cross validation.** The percentage of each moiety with correct, excess, and incorrect predicted ionization states for various pK_a_ precision factors (stdev).

|  |  | stdev: 0.0 | stdev: 1.0 | stdev: 1.5 | stdev: 2.0 |
| --- | --- | --- | --- | --- | --- |
| Alcohol | Correct (%)  Excess (%)  Incorrect (%) | 100.0 ± 0.0  0.0 ± 0.0  0.0 ± 0.0 | 100.0 ± 0.0  0.0 ± 0.0  0.0 ± 0.0 | 100.0 ± 0.0  0.0 ± 0.0  0.0 ± 0.0 | 100.0 ± 0.0  0.0 ± 0.0  0.0 ± 0.0 |
| Amide | Correct (%)  Excess (%)  Incorrect (%) | 88.9 ± 7.9  0.0 ± 0.0  11.1 ± 7.9 | 88.9 ± 7.9  0.0 ± 0.0  11.1 ± 7.9 | 88.9 ± 7.9  0.0 ± 0.0  11.1 ± 7.9 | 55.6 ± 39.3  33.3 ± 47.1  11.1 ± 7.9 |
| Amide_electronegative | Correct (%)  Excess (%)  Incorrect (%) | 36.1 ± 2.0  43.1 ± 30.5  20.8 ± 29.5 | 40.3 ± 7.1  59.7 ± 7.1  0.0 ± 0.0 | 40.3 ± 7.1  59.7 ± 7.1  0.0 ± 0.0 | 40.3 ± 7.1  59.7 ± 7.1  0.0 ± 0.0 |
| AmidineGuanidine1 | Correct (%)  Excess (%)  Incorrect (%) | 100.0 ± 0.0  0.0 ± 0.0  0.0 ± 0.0 | 100.0 ± 0.0  0.0 ± 0.0  0.0 ± 0.0 | 100.0 ± 0.0  0.0 ± 0.0  0.0 ± 0.0 | 100.0 ± 0.0  0.0 ± 0.0  0.0 ± 0.0 |
| AmidineGuanidine2 | Correct (%)  Excess (%)  Incorrect (%) | 100.0 ± 0.0  0.0 ± 0.0  0.0 ± 0.0 | 100.0 ± 0.0  0.0 ± 0.0  0.0 ± 0.0 | 100.0 ± 0.0  0.0 ± 0.0  0.0 ± 0.0 | 100.0 ± 0.0  0.0 ± 0.0  0.0 ± 0.0 |
| Amines_primary_secondary_tertiary | Correct (%)  Excess (%)  Incorrect (%) | 94.9 ± 1.2  0.0 ± 0.0  5.1 ± 1.2 | 94.9 ± 1.2  0.0 ± 0.0  5.1 ± 1.2 | 94.9 ± 1.2  0.0 ± 0.0  5.1 ± 1.2 | 1.9 ± 0.9  98.1 ± 0.9  0.0 ± 0.0 |
| Anilines_primary | Correct (%)  Excess (%)  Incorrect (%) | 66.2 ± 5.0  0.0 ± 0.0  33.8 ± 5.0 | 25.9 ± 3.4  74.1 ± 3.4  0.0 ± 0.0 | 25.9 ± 3.4  74.1 ± 3.4  0.0 ± 0.0 | 25.9 ± 3.4  74.1 ± 3.4  0.0 ± 0.0 |
| Anilines_secondary | Correct (%)  Excess (%)  Incorrect (%) | 77.3 ± 14.9  0.0 ± 0.0  22.7 ± 14.9 | 8.3 ± 11.8  91.7 ± 11.8  0.0 ± 0.0 | 8.3 ± 11.8  91.7 ± 11.8  0.0 ± 0.0 | 8.3 ± 11.8  91.7 ± 11.8  0.0 ± 0.0 |
| Anilines_tertiary | Correct (%)  Excess (%)  Incorrect (%) | 61.4 ± 5.0  0.0 ± 0.0  38.6 ± 5.0 | 31.6 ± 8.6  68.4 ± 8.6  0.0 ± 0.0 | 31.6 ± 8.6  68.4 ± 8.6  0.0 ± 0.0 | 31.6 ± 8.6  68.4 ± 8.6  0.0 ± 0.0 |
| Aromatic_nitrogen_protonated | Correct (%)  Excess (%)  Incorrect (%) | 87.5 ± 0.0  0.0 ± 0.0  12.5 ± 0.0 | 87.5 ± 0.0  0.0 ± 0.0  12.5 ± 0.0 | 87.5 ± 0.0  0.0 ± 0.0  12.5 ± 0.0 | 8.3 ± 5.9  91.7 ± 5.9  0.0 ± 0.0 |
| Aromatic_nitrogen_unprotonated | Correct (%)  Excess (%)  Incorrect (%) | 64.2 ± 3.7  0.0 ± 0.0  35.8 ± 3.7 | 29.4 ± 6.7  70.6 ± 6.7  0.0 ± 0.0 | 29.4 ± 6.7  70.6 ± 6.7  0.0 ± 0.0 | 29.4 ± 6.7  70.6 ± 6.7  0.0 ± 0.0 |
| Carboxyl | Correct (%)  Excess (%)  Incorrect (%) | 43.3 ± 7.5  41.3 ± 29.2  15.4 ± 21.7 | 38.3 ± 0.5  61.7 ± 0.5  0.0 ± 0.0 | 38.3 ± 0.5  61.7 ± 0.5  0.0 ± 0.0 | 38.3 ± 0.5  61.7 ± 0.5  0.0 ± 0.0 |
| Imide | Correct (%)  Excess (%)  Incorrect (%) | 33.3 ± 47.1  66.7 ± 47.1  0.0 ± 0.0 | 33.3 ± 47.1  66.7 ± 47.1  0.0 ± 0.0 | 33.3 ± 47.1  66.7 ± 47.1  0.0 ± 0.0 | 33.3 ± 47.1  66.7 ± 47.1  0.0 ± 0.0 |
| Imide2 | Correct (%)  Excess (%)  Incorrect (%) | 100.0 ± 0.0  0.0 ± 0.0  0.0 ± 0.0 | 100.0 ± 0.0  0.0 ± 0.0  0.0 ± 0.0 | 100.0 ± 0.0  0.0 ± 0.0  0.0 ± 0.0 | 100.0 ± 0.0  0.0 ± 0.0  0.0 ± 0.0 |
| N-hydroxyamide | Correct (%)  Excess (%)  Incorrect (%) | 100.0 ± 0.0  0.0 ± 0.0  0.0 ± 0.0 | 100.0 ± 0.0  0.0 ± 0.0  0.0 ± 0.0 | 100.0 ± 0.0  0.0 ± 0.0  0.0 ± 0.0 | 100.0 ± 0.0  0.0 ± 0.0  0.0 ± 0.0 |
| O=C-C=C-OH | Correct (%)  Excess (%)  Incorrect (%) | 53.3 ± 24.9  26.7 ± 37.7  20.0 ± 16.3 | 26.7 ± 9.4  73.3 ± 9.4  0.0 ± 0.0 | 26.7 ± 9.4  73.3 ± 9.4  0.0 ± 0.0 | 26.7 ± 9.4  73.3 ± 9.4  0.0 ± 0.0 |
| Peroxide1 | Correct (%)  Excess (%)  Incorrect (%) | 100.0 ± 0.0  0.0 ± 0.0  0.0 ± 0.0 | 100.0 ± 0.0  0.0 ± 0.0  0.0 ± 0.0 | 100.0 ± 0.0  0.0 ± 0.0  0.0 ± 0.0 | 100.0 ± 0.0  0.0 ± 0.0  0.0 ± 0.0 |
| Peroxide2 | Correct (%)  Excess (%)  Incorrect (%) | 100.0 ± 0.0  0.0 ± 0.0  0.0 ± 0.0 | 100.0 ± 0.0  0.0 ± 0.0  0.0 ± 0.0 | 100.0 ± 0.0  0.0 ± 0.0  0.0 ± 0.0 | 100.0 ± 0.0  0.0 ± 0.0  0.0 ± 0.0 |
| Phenol | Correct (%)  Excess (%)  Incorrect (%) | 86.3 ± 2.7  0.0 ± 0.0  13.7 ± 2.7 | 86.3 ± 2.7  0.0 ± 0.0  13.7 ± 2.7 | 3.7 ± 0.8  96.3 ± 0.8  0.0 ± 0.0 | 3.7 ± 0.8  96.3 ± 0.8  0.0 ± 0.0 |
| Phenyl_carboxyl | Correct (%)  Excess (%)  Incorrect (%) | 35.5 ± 14.5  49.6 ± 35.3  14.9 ± 21.1 | 29.1 ± 6.1  70.9 ± 6.1  0.0 ± 0.0 | 29.1 ± 6.1  70.9 ± 6.1  0.0 ± 0.0 | 29.1 ± 6.1  70.9 ± 6.1  0.0 ± 0.0 |
| Phenyl_thiol | Correct (%)  Excess (%)  Incorrect (%) | 75.6 ± 17.5  0.0 ± 0.0  24.4 ± 17.5 | 12.2 ± 8.7  87.8 ± 8.7  0.0 ± 0.0 | 12.2 ± 8.7  87.8 ± 8.7  0.0 ± 0.0 | 12.2 ± 8.7  87.8 ± 8.7  0.0 ± 0.0 |
| Phosphate | Correct (%)  Excess (%)  Incorrect (%) | 81.5 ± 10.5  18.5 ± 10.5  0.0 ± 0.0 | 81.5 ± 10.5  18.5 ± 10.5  0.0 ± 0.0 | 81.5 ± 10.5  18.5 ± 10.5  0.0 ± 0.0 | 81.5 ± 10.5  18.5 ± 10.5  0.0 ± 0.0 |
| Phosphate_diester | Correct (%)  Excess (%)  Incorrect (%) | 26.2 ± 10.3  73.8 ± 10.3  0.0 ± 0.0 | 26.2 ± 10.3  73.8 ± 10.3  0.0 ± 0.0 | 26.2 ± 10.3  73.8 ± 10.3  0.0 ± 0.0 | 26.2 ± 10.3  73.8 ± 10.3  0.0 ± 0.0 |
| Phosphinic_acid | Correct (%)  Excess (%)  Incorrect (%) | 84.1 ± 13.7  15.9 ± 13.7  0.0 ± 0.0 | 84.1 ± 13.7  15.9 ± 13.7  0.0 ± 0.0 | 84.1 ± 13.7  15.9 ± 13.7  0.0 ± 0.0 | 84.1 ± 13.7  15.9 ± 13.7  0.0 ± 0.0 |
| Phosphonate | Correct (%)  Excess (%)  Incorrect (%) | 74.5 ± 7.3  25.5 ± 7.3  0.0 ± 0.0 | 74.5 ± 7.3  25.5 ± 7.3  0.0 ± 0.0 | 74.5 ± 7.3  25.5 ± 7.3  0.0 ± 0.0 | 74.5 ± 7.3  25.5 ± 7.3  0.0 ± 0.0 |
| Phosphonate_ester | Correct (%)  Excess (%)  Incorrect (%) | 92.6 ± 10.5  7.4 ± 10.5  0.0 ± 0.0 | 92.6 ± 10.5  7.4 ± 10.5  0.0 ± 0.0 | 92.6 ± 10.5  7.4 ± 10.5  0.0 ± 0.0 | 92.6 ± 10.5  7.4 ± 10.5  0.0 ± 0.0 |
| Primary_hydroxyl_aminePrimary_hydroxyl_amine | Correct (%)  Excess (%)  Incorrect (%) | 88.9 ± 15.7  0.0 ± 0.0  11.1 ± 15.7 | 22.2 ± 31.4  66.7 ± 47.1  11.1 ± 15.7 | 22.2 ± 31.4  66.7 ± 47.1  11.1 ± 15.7 | 22.2 ± 31.4  66.7 ± 47.1  11.1 ± 15.7 |
| Ringed_imide1 | Correct (%)  Excess (%)  Incorrect (%) | 100.0 ± 0.0  0.0 ± 0.0  0.0 ± 0.0 | 100.0 ± 0.0  0.0 ± 0.0  0.0 ± 0.0 | 100.0 ± 0.0  0.0 ± 0.0  0.0 ± 0.0 | 100.0 ± 0.0  0.0 ± 0.0  0.0 ± 0.0 |
| Ringed_imide2 | Correct (%)  Excess (%)  Incorrect (%) | 100.0 ± 0.0  0.0 ± 0.0  0.0 ± 0.0 | 100.0 ± 0.0  0.0 ± 0.0  0.0 ± 0.0 | 100.0 ± 0.0  0.0 ± 0.0  0.0 ± 0.0 | 100.0 ± 0.0  0.0 ± 0.0  0.0 ± 0.0 |
| Sulfate | Correct (%)  Excess (%)  Incorrect (%) | 100.0 ± 0.0  0.0 ± 0.0  0.0 ± 0.0 | 100.0 ± 0.0  0.0 ± 0.0  0.0 ± 0.0 | 100.0 ± 0.0  0.0 ± 0.0  0.0 ± 0.0 | 100.0 ± 0.0  0.0 ± 0.0  0.0 ± 0.0 |
| Sulfinic_acid | Correct (%)  Excess (%)  Incorrect (%) | 66.7 ± 0.0  33.3 ± 0.0  0.0 ± 0.0 | 66.7 ± 0.0  33.3 ± 0.0  0.0 ± 0.0 | 66.7 ± 0.0  33.3 ± 0.0  0.0 ± 0.0 | 66.7 ± 0.0  33.3 ± 0.0  0.0 ± 0.0 |
| Sulfonamide | Correct (%)  Excess (%)  Incorrect (%) | 95.7 ± 3.0  0.0 ± 0.0  4.3 ± 3.0 | 95.7 ± 3.0  0.0 ± 0.0  4.3 ± 3.0 | 95.7 ± 3.0  0.0 ± 0.0  4.3 ± 3.0 | 95.7 ± 3.0  0.0 ± 0.0  4.3 ± 3.0 |
| Sulfonate | Correct (%)  Excess (%)  Incorrect (%) | 100.0 ± 0.0  0.0 ± 0.0  0.0 ± 0.0 | 100.0 ± 0.0  0.0 ± 0.0  0.0 ± 0.0 | 100.0 ± 0.0  0.0 ± 0.0  0.0 ± 0.0 | 66.7 ± 47.1  33.3 ± 47.1  0.0 ± 0.0 |
| Thioic_acid | Correct (%)  Excess (%)  Incorrect (%) | 61.1 ± 28.3  0.0 ± 0.0  38.9 ± 28.3 | 27.8 ± 20.8  50.0 ± 40.8  22.2 ± 31.4 | 38.9 ± 28.3  61.1 ± 28.3  0.0 ± 0.0 | 38.9 ± 28.3  61.1 ± 28.3  0.0 ± 0.0 |
| Thiol | Correct (%)  Excess (%)  Incorrect (%) | 100.0 ± 0.0  0.0 ± 0.0  0.0 ± 0.0 | 100.0 ± 0.0  0.0 ± 0.0  0.0 ± 0.0 | 100.0 ± 0.0  0.0 ± 0.0  0.0 ± 0.0 | 100.0 ± 0.0  0.0 ± 0.0  0.0 ± 0.0 |
| Vinyl_alcohol | Correct (%)  Excess (%)  Incorrect (%) | 100.0 ± 0.0  0.0 ± 0.0  0.0 ± 0.0 | 100.0 ± 0.0  0.0 ± 0.0  0.0 ± 0.0 | 100.0 ± 0.0  0.0 ± 0.0  0.0 ± 0.0 | 100.0 ± 0.0  0.0 ± 0.0  0.0 ± 0.0 |
| Average | Correct (%)  Excess (%)  Incorrect (%) | 79.9  11.2  9.0 | 69.6  28.2  2.2 | 67.6  31.2  1.2 | 60.9  38.3  0.7 |
|  |  |  |  |  |  |
|  |  |  |  |  |  |
